# Positive associations between upregulated levels of stress-induced phosphoprotein 1 and matrix metalloproteinase-9 in endometriosis/adenomyosis

**DOI:** 10.1371/journal.pone.0190573

**Published:** 2018-01-05

**Authors:** Hsin-Shih Wang, Chia-Lung Tsai, Pi-Yueh Chang, Angel Chao, Ren-Chin Wu, Shun-Hua Chen, Chin-Jung Wang, Chih-Feng Yen, Yun-Shien Lee, Tzu-Hao Wang

**Affiliations:** 1 Department of Obstetrics and Gynecology, LinKou Medical Center, Chang Gung Memorial Hospital and Chang Gung University, Taoyuan, Taiwan; 2 Genomic Medicine Research Core Laboratory, Chang Gung Memorial Hospital, Taoyuan, Taiwan; 3 Department of Laboratory Medicine, Chang Gung Memorial Hospital, Taoyuan, Taiwan; 4 Gynecologic Cancer Research Centre, LinKou Medical Center, Chang Gung Memorial Hospital, Taoyuan Taiwan; 5 Department of Clinical Pathology, Chang Gung Memorial Hospital, Taoyuan, Taiwan; 6 Graduate Institutes of Biomedical Sciences, College of Medicine, Chang Gung University, Taoyuan, Taiwan; 7 Department of Biotechnology, Ming-Chuan University, Taoyuan, Taiwan; National Institute for Research in Reproductive Health, INDIA

## Abstract

Stress-induced phosphoprotein-1 (STIP1), an adaptor protein that coordinates the functions of HSP70 and HSP90 in protein folding, has been implicated in the development of human gynecologic malignancies. This case-control study investigates STIP1 serum levels and tissue expression in relation to endometriosis/adenomyosis in Taiwanese population. Female patients with surgically confirmed endometriosis/adenomyosis were compared with women free of endometriosis/adenomyosis. Serum STIP1 levels were measured using an enzyme-linked immunosorbent assay and surgical tissues were analyzed by immunohistochemistry. Both epithelial and stromal cells in surgical tissues of endometriosis and adenomyosis expressed STIP1 and MMP-9. Notably, MMP-9 expression was significantly decreased when STIP1 expression was knocked-down. *In vitro* experiments revealed that STIP1 was capable of binding to the MMP-9 promoter and enhanced its transcriptional expression. The preoperative serum STIP1 levels of patients with endometriosis/adenomyosis were significantly higher than those of the controls. In brief, our data suggest an association between STIP1 levels and endometriosis/adenomyosis.

## Introduction

Endometriosis has a significant impact on public health, with an estimated prevalence of 5%−10% in women of reproductive age [1]. Owing to the presence and growth of ectopic endometrial tissues outside the endometrial cavity, this condition has a profound effect on women’s quality of life [2] and reproductive potential [3]. Although the pathogenesis of endometriosis remains controversial and poorly understood [4,5], it is widely accepted that genetic factors [6–8] and immune dysfunction [1,4,5] contribute to its development.

Stress-induced phosphoprotein (STIP1, Gene ID 10963; HPRD 05454)—a 62.6 kDa protein also known as heat shock protein (HSP)-organizing protein (HOP) [9]—is an adaptor protein that coordinates the functions of HSP70 and HSP90 in protein folding. Increased STIP1 expression is involved in the pathogenesis of gynecologic malignancies, including ovarian [10–13] and endometrial [14] cancer. Evidence indicates that STIP1 can stimulate DNA synthesis and enhance cell proliferation [10]. The knockdown of STIP1 expression in cancer cells has been shown to reduce tumor invasiveness through downregulation of matrix metalloproteinases (MMPs) [15].

Extracellular matrix remodeling has been postulated to play a role in the pathogenesis of endometriosis [16,17]. Increased levels and activity of MMP-9 have been reported, especially at advanced stages of the disease [17]. In addition, MMP-9 concentrations are higher in the plasma and peritoneal fluids of patients with endometriosis than in those of healthy women [18]. In light of these findings, MMP-9 could be involved in the tissue growth and breakdown cycle that occurs during endometriosis [19], thereby potentially affecting the migration and dissemination of endometrial cells outside the uterus.

STIP1 is thought to regulate the expression of MMPs [15]; moreover, endometriosis shares certain features with cancers [19] in which STIP1 has been shown to play a role [10–14]. Therefore, this study tested the hypothesis that STIP is involved in the pathogenesis of endometriosis through various experimental approaches. Specifically, we designed the current case–control study to investigate serum STIP1 levels and tissue expression in relation to endometriosis in a Taiwanese population. We also investigated the effect of STIP1 knockdown on MMP-9 expression and activity in endometrial cancer cell lines. Finally, we examined the molecular mechanisms by which STIP1 regulates MMP-9 at the transcriptional level.

## Materials and methods

### Ethics statement

All procedures were conducted in compliance with the tenets of the Declaration of Helsinki and were approved by the Institutional Review Board of Chang Gung Memorial Hospital (IRB approval #94-975B, #98-1982B, and #98-1995A3). Written informed consent was obtained from the enrolled patients.

### Serum collection

Although the presence of an identical etiology for adenomyosis and endometriosis remains controversial, the diseases share characteristics such as clinical impacts, elevated CA125 levels, ectopic endometrial tissues, and responses to hormonal treatments [20,21]. Hence, both diseases were included in this study. The serum CA125 level is elevated during menstruation, and is therefore commonly assayed 1 week after the end of menses. Serum STIP1 was assayed in the sera collected for CA125 measurement. In this study, the study group consisted of patients with clinically confirmed endometriosis/adenomyosis, whom were treated either by laparotomy or laparoscopy at Chang Gung Memorial Hospital. Control sera were collected during routine health surveys from women who were confirmed to be free of endometriosis/adenomyosis and without a history of dysmenorrhea.

### Culture and treatment of cell lines

Human endometrial cancer cells (RL95-2) and endometrioid ovarian cancer cells (MDAH2774) were obtained from the American Type Culture Collection (Manassas, VA, USA). The human endometrial cancer cell line ARK2 was kindly provided by Dr. Alessandro D. Santin (Yale University School of Medicine, New Haven, CT, USA). Both RL95-2 and MDAH2774 cells were cultured in Dulbecco’s modified Eagle’s medium (DMEM)/F12, whereas ARK2 cells were maintained in RPMI medium containing 10% fetal bovine serum with penicillin (100U/ml) and streptomycin (100U/ml). In the functional experiments, cells were pretreated with one of the following inhibitors: the histone deacetylase (HDAC) inhibitor trichostatin A (TSA; Sigma-Aldrich, St. Louds, MO) at a concentration of 20 nM for 48 h; the DNA methyltransferase (DNMT) inhibitor 5-aza-2’-deoxycytidine (5’-AZA; Sigma-Aldrich, St. Louds, MO) at a concentration of 10 μM for 24 h; the proteasome inhibitor MG-132 (Sigma-Aldrich, St. Louds, MO) at a concentration of 25 μM for 5 h; or the JAK2 inhibitor AG490 (Merck, Kenilworth, NJ) at a concentration of 35 μM for 5 h.

### DNA constructions

MMP-9 promoter constructs -940, -710, -580, -166, -66, -73mutAP1, -533mutAP1 and -73/-533 mutAP1 were generously provided by Dr. Yao Chang (National Health Research Institutes, Tainan, Taiwan) [22]. The MMP-9 promoter NF-kB and EGR1 mutants (mNF-kB, mEGR1) were kindly gifted by Dr. Young Han Lee (Konkuk University, Seoul, Korea) [23]. All the aforementioned promoter constructs contained DNA regions localized from −1 kb to +137 bp in the MMP-9 promoter. The −2 kb MMP-9 promoter construct was amplified from human genomic DNA using the following MMP-9 primers: forward, 5’-CTCGGCGGCCAAGCTTACGGTGCTTGACACAGTAAATC-3’ and reverse, 5’-CCGGATTGCCAAGCTTGGTGAGGGCAGAGGTGTCT-3’. The polymerase chain reaction (PCR) conditions used were as follows: initial denaturation at 95°C for 3 min, followed by 40 cycles of 30-s denaturation at 95°C, annealing for 30 s at 55°C, and extension for 90 s at 72°C, with a final extension at 72°C for 10 min. The PCR products underwent spin column purification and were subsequently mixed with a *HindIII* (MMP-9)-treated pGL4.2 vector (Promega, Madison, WI, USA) by using an In-Fusion HD cloning kit (Clontech, Mountain View, CA, USA). DNA sequences were confirmed with an ABI DNA autosequencer (Applied Biosystems, Foster City, CA, USA).

### Transfection of small interfering (si) RNA

ARK2 cells (3 × 10^6^ cells per 10-cm dish) were transfected with double-stranded RNA (50 nM) in Lipofectamine RNAimax (Invitrogen, Carlsbad, CA, USA) according to the manufacturer’s protocol. Small interfering (si)-STIP1 and control siRNA were purchased from Sigma Aldrich. At 72 h post-transfection, real-time (RT) quantitative PCR (QPCR) and western blot analyses were used to confirm the suppression of gene expression.

### Wound closure assay

ARK2 cells were seeded on culture inserts (Ibidi GmbH, Planegg, Germany) at a density of 1 × 10^4^ cells per well in complete culture medium. After an overnight culture, the culture inserts were removed to create cell-free gaps and the medium was replaced with fresh serum-free medium. Phase-contrast images of the gaps were obtained at 0 and 20 h of incubation using an inverted microscope (10× magnification) and quantified with WimScratch (Ibidi GmbH).

### RNA extraction and RT QPCR

Total RNA was isolated with TRIzol reagent (Invitrogen, Carlsbad, CA, USA). First-strand cDNA was synthesized with an oligo-T primer using a Superscript III First Strand Synthesis kit (Invitrogen, Carlsbad, CA, USA). SYBR Green gene expression assay was used to quantify STIP1 and MMP-9 expression levels. The following primers were used for STIP1: forward, 5’-ACTAACATGACTTACATTACC-3’, and reverse, 5’-ATATGCTTTGGCAATCTG-3’. The primers used for MMP-9 were as follows: forward, 5’-CTTCACTTTCCTGGGTAA-3’, and reverse, 5’-ACAAACTGTATCCTTGGT-3’. The following conditions were used for the QPCR: initial step at 95°C for 10 min, followed by 40 cycles of 10 s at 95°C, 15 s at 60°C, and 10 s at 72°C.

### Western blot analysis

Cell lysates were prepared with radioimmunoprecipitation assay buffer (150 mM NaCl, 20 mM Tris-Cl pH7.5, 1% Triton X-100, 1% NP40, 0.1% SDS, 0.5% deoxycholate) containing freshly added proteinase and phosphatase inhibitors (Bionovas, Toronto, Canada). Protein concentrations were assayed using the Bradford method. One hundred micrograms of each sample were subjected to electrophoresis in 10% SDS-polyacrylamide gels and subsequently transferred onto nitrocellulose membranes. All antibodies were obtained from the following commercial sources: MMP-9 (Cell Signaling Technology, Danvers, MA, USA), calmodulin binding peptide tag (CBP; Millipore, Billerica, MA, USA), halo tag (Promega, Madison, WI, USA), actin and STIP1 (Santa Cruz Biotechnology, Santa Cruz, CA, USA), and corresponding horseradish peroxidase-conjugated antibodies (Santa Cruz Biotechnology). Enhanced chemiluminescence reagents were obtained from Millipore. The autoradiogram signal intensity was quantified with the UN-SCAN-IT graph digitizing software (Silk Scientific, Orem, UT, USA); the relative intensity of each sample was normalized against the corresponding actin intensity.

### Cell fractionation

After trypsinization and washing with cold PBS, the cells were resuspended in hypotonic buffer (20 mM Tris-HCl, pH 7.4, 10 mM NaCl, 3 mM MgCl_2_, 10% NP-40). The supernatant containing the cytoplasmic fraction was subsequently collected by centrifugation, and the remaining nuclear pellets were lysed with a cell extraction buffer (100 mM Tris-HCl, pH 7.4, 2 mM Na_3_VO_4_, 100 mM NaCl, 1% triton X-100, 1 mM EDTA, 10% glycerol, 1 mM EGTA, 0.1% SDS, 1 mM NaF, 0.5% deoxycholate, and 20 mM Na_4_P_2_O_7_).

### Chromatin immunoprecipitation assay

Cells were treated with 1% formaldehyde at room temperature for 10 min to cross-link the proteins to the DNA. The reaction was stopped by adding glycine (0.125 M), following which the cells were scraped from the culture dishes into PBS containing proteinase inhibitors. The pellets were resuspended in lysis buffer (5 mM PIPES/KOH pH 8.0, 85 mM KCL, 0.5% NP-40) and left on ice for 10 min. Nuclei collected by centrifugation were lysed with a nuclear lysis buffer (50 mM Tris pH 8.1, 10 mM EDTA, 1% SDS) and incubated on ice for 10 min. Lysates were sonicated to obtain chromatin (mean length, approximately 600 bp). After centrifugation, 1 mg of the protein supernatant was diluted (5-fold) in chromatin immunoprecipitation (ChIP) dilution buffer (0.01% SDS, 1.1% Triton X-100, 1.2 mM EDTA, 16.7 mM Tris pH 8.1, 167 mM NaCl). Immunocomplexes were precipitated overnight at 4°C with 5 μg of specific antibodies, followed by four sequential washes with low salt, high salt, LiCl, and Tris-EDTA buffer (pH 7.8). The washed immunoprecipitate was then eluted in elution buffer (1% SDS, 0.1 M NaHCO3) at room temperature and subjected to two 15-min runs of vortex and centrifugation. Formaldehyde cross-links were reversed by incubation in a water bath at 65°C for 5 h. After treatment with proteinase K and subsequent phenol/chloroform extraction, DNA fragments were recovered by ethanol precipitation and amplified by QPCR or PCR. The primers used to detect MMP-9 promoter were as follows: 1F, 5’-TGCTTGACACAGTAAATC-3’; 1R, 5’-GAGATGGAGATAATTAACTTC-3’; 2F, 5’-CATCTCACAGTCTCATTT-3’; 2R, 5’-CGAGTAGCTGGTATTATAG-3’; 3F, 5’-CCTATAATACCAGCTACTC-3’; 3R, 5’-TCTATATTCACCTTCTTCAA-3’; 4F, 5’-TTGAAGAAGGTGAATATAGA-3’; 4R, 5’-TGTAACCTGGAGTAAATG-3’; 5F, 5’-GCAGTTGAAGAATCCTAA-3’; 5R, 5’-CATTCCTGTAATCTTAGCA-3’. The following conditions for QPCR were used: initial step at 95°C for 10 min, followed by 40 cycles of 10 s at 95°C, 15 s at 60°C, and 10 s at 72°C. The PCR conditions were as follows: 3 min at 95°C followed by 30 cycles of 15 s at 95°C, 15 s at 50°C, and 15 s at 72°C. The PCR products were separated in 8% polyacrylamide gels. The STIP1 antibodies were purchased from Abnova (Taipei, Taiwan) and Santa Cruz Biotechology.

### Zymography

MMP-9 activity was measured by zymography as described previously [23]. In brief, culture medium supernatant was concentrated using a Centricon YM50 filter device (Millipore) and separated in SDS-PAGE containing 1% gelatin under nonreducing conditions. After washing with 2.5% triton X-100 and an overnight incubation in a development buffer, the gel was stained with 0.25% Coomassie Brilliant Blue R-250 (Bio-Rad, Hercules, CA, USA) and subsequently destained in a staining solution without dye. MMP-9 activity was observed as clear bands against the blue background of the gelatin gel.

### Measurement of serum STIP1 levels with enzyme-linked immunosorbent assay

The development of the enzyme-linked immunosorbent assay (ELISA) for quantifying serum STIP1 levels has been described previously [10]. Briefly, 96-well plates (Nunc F8 MaxiSorp, A/S, Roskilde, Denmark) were coated with a mouse monoclonal capture antibody (Abnova). A second biotinylated mouse monoclonal antibody (Abnova) was utilized for detection. Streptavidin was coupled with peroxidase to use the conjugate as a signal generator. After washing, tetramethylbenzidine substrate solution was added, and the reaction was stopped with 2N H_2_SO_4_ (100 μL per well). Optical density was measured at 450 nm using a 96-well microplate reader (Spectra Max model 190; Molecular Devices, Sunnyvale, CA, USA). The minimum detection limit was 2 ng/mL. The intra-assay coefficients of variation were 4.6% at 59.5 ng/mL (n = 6) and 5.6% at 16.5 ng/mL (n = 6), respectively. The interassay coefficients of variation were 10.6% at 59.5 ng/mL (n = 7) and 9.4% at 16.5 ng/mL (n = 7).

### Immunohistochemistry

Formalin-fixed paraffin-embedded (FFPE) endometriosis and adenomyosis tissues from the tissue bank were sectioned (thickness, 4 μm) and deparaffinized with xylene. After rehydration in a series of graded ethanol solutions, the sections were stained with anti-human STIP1 (Abnova) and anti-MMP9 (Abcam, #ab76003, Cambridge, UK). Hematoxylin was used for counterstaining.

## Results

### Serum STIP1 levels were higher in patients with endometriosis

**Fig 1:**
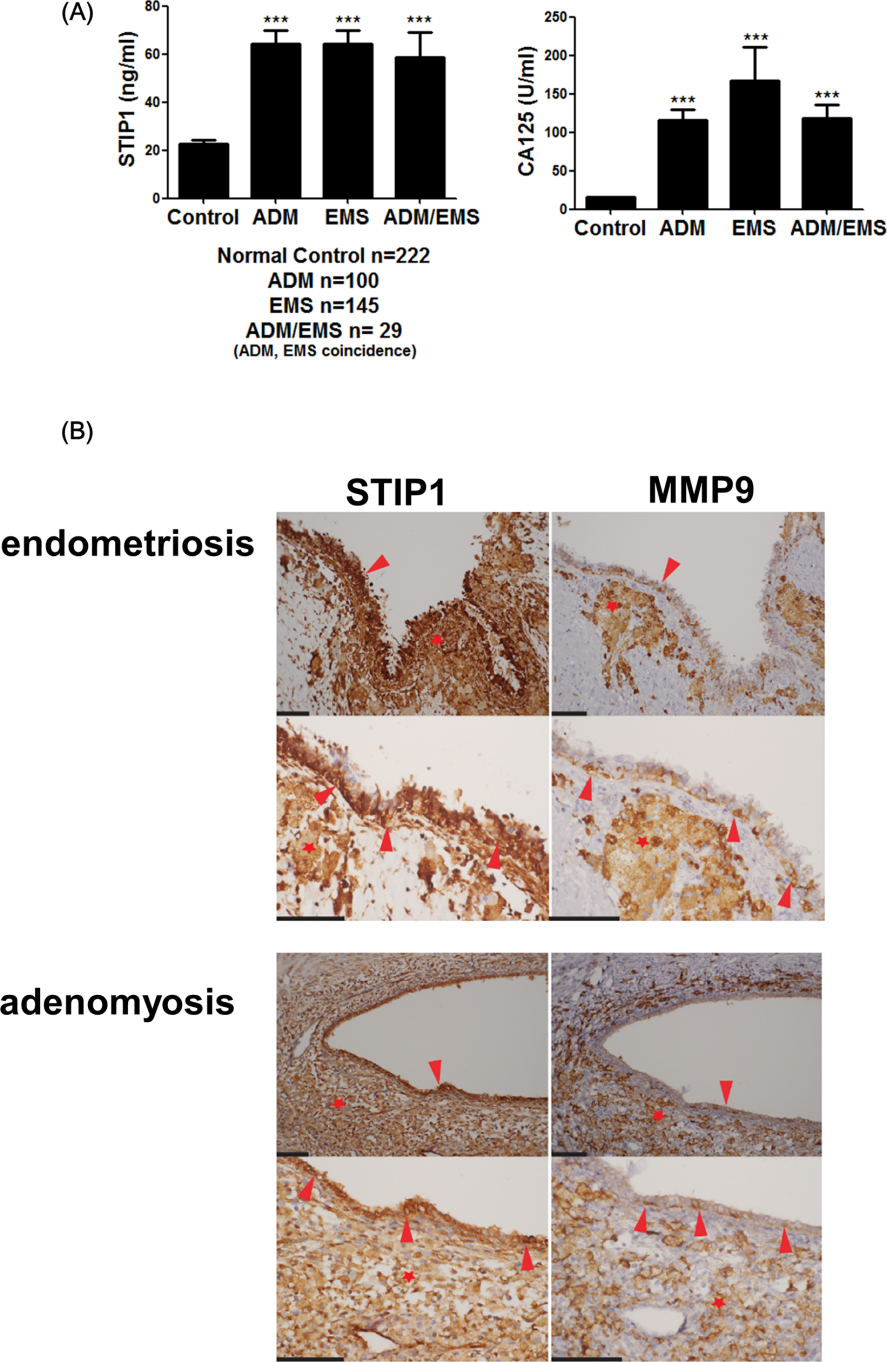
STIP1 overexpression in endometriosis/adenomyosis. (A) Serum levels of STIP1 (left panel) and CA125 (right panel) were significantly higher in patients with endometriosis (EMS), adenomyosis (ADM), or coexisting endometriosis and adenomyosis (EMS/ADM) than in the age-matched controls. Statistical differences were calculated with Student’s *t*-test. *p < 0.05 and **p < 0.01. (B) Photomicrographs of immunohistochemical staining for STIP1 and MMP9 in a representative case of ovarian endometriosis and uterine adenomyosis taken with a 20× objective (upper panels) and 40× objective (lower panels) lens. Both ectopic endometrial epithelia (arrowheads) and stroma (stars) showed cytoplasmic expressions of STIP1 and MMP9, in both endometriosis and adenomyosis. Scale bars represent 100 μm. Abbreviations: EMS, endometriosis; ADM, adenomyosis.

Serum STIP1 levels were measured in 222 patients with pathologically-proven endometriosis and/or adenomyosis (mean age, 40.1 ± 7.7 years) and 222 age-matched control women (mean age, 40.1 ± 7.7 years). Preoperative serum STIP1 levels in patients with endometriosis/adenomyosis were significantly higher than those in the controls (64.3 ± 5.5 ng/mL in the adenomyosis group, 64.4 ± 5.6 ng/mL in the endometriosis group, and 68.6 ± 10.5 ng/mL in the coexisting adenomyosis and endometriosis groups *vs* 22.8 ± 1.3 ng/mL in the controls, P < 0.001). Similarly, preoperative serum CA125 levels were significantly higher in patients with endometriosis than in controls (116.4 ± 13.3 ng/mL in the adenomyosis group, 166.5 ± 44.6 ng/mL in the endometriosis group, and 118.8 ± 16.6 ng/mL in the coexisting adenomyosis and endometriosis group *vs* 15.7 ± 0.5 ng/mL in the controls, P < 0.001; (Fig 1A). Receiver operating characteristic (ROC) curve analysis revealed that the cutoff point that maximized both the sensitivity and specificity of serum STIP1 levels in the detection of endometriosis was 51.2 ng/mL (specificity, 79.3%; sensitivity, 53.6%; Table 1). A cutoff value of 98.6 ng/mL for serum STIP1 levels had a 95% specificity for the diagnosis of endometriosis, although the sensitivity dropped to 18.9%. Serum CA125 levels were superior to STIP1 levels in the detection of endometriosis (specificity, 95.0%; sensitivity, 75.7%; Table 1). The presence of both serum STIP1 > 51.2 ng/mL and CA125 > 35 U/mL had 100% specificity for endometriosis, but sensitivity was as low as 39.2%. Conversely, the presence of either STIP1 > 51.2 ng/mL or CA125 > 35 U/mL had lower specificity (78.4%) and a sensitivity of 90.1% (Table 1).

**Table 1:** Sensitivity, specificity, positive and negative predictive values, and accuracy of serum STIP1 and CA125 levels in the detection of endometriosis.

	Sensitivity (%)	Specificity (%)	PPV (%)	NPV (%)
STIP1 >51.2 ng/mL	53.6	79.3	72.1	36.9
STIP1 > 98.6 ng/mL	18.9	95.0	79.2	54.0
CA125 >35 U/mL	75.7	95.0	98.8	80.2
STIP1 >51.2 ng/mL and CA125 >35 U/mL	39.2	100	100	62.2
STIP1 >51.2 ng/mL or CA125 >35 U/mL	90.1	78.4	80.6	88.8

Abbreviations: PPV, positive predictive value; NPV, negative predictive value

### Immunohistochemistry

Immunostaining for STIP1 was markedly positive in both ovarian endometriosis and adenomyosis. In serial sections obtained from the same tissues, both epithelial and stromal cells of endometriosis/adenomyosis expressed STIP1 and MMP9 (Fig 1B). In both secretory and proliferative normal endometrial tissues (S1 Fig), positive immunostaining for STIP1 and nearly negative staining for MMP9 was noted. These results suggest that other factors, in addition to STIP1, may be required for the stimulation of MMP9 in endometriosis/adenomyosis.

### STIP1 stimulates MMP-9 expression

**Fig 2:**
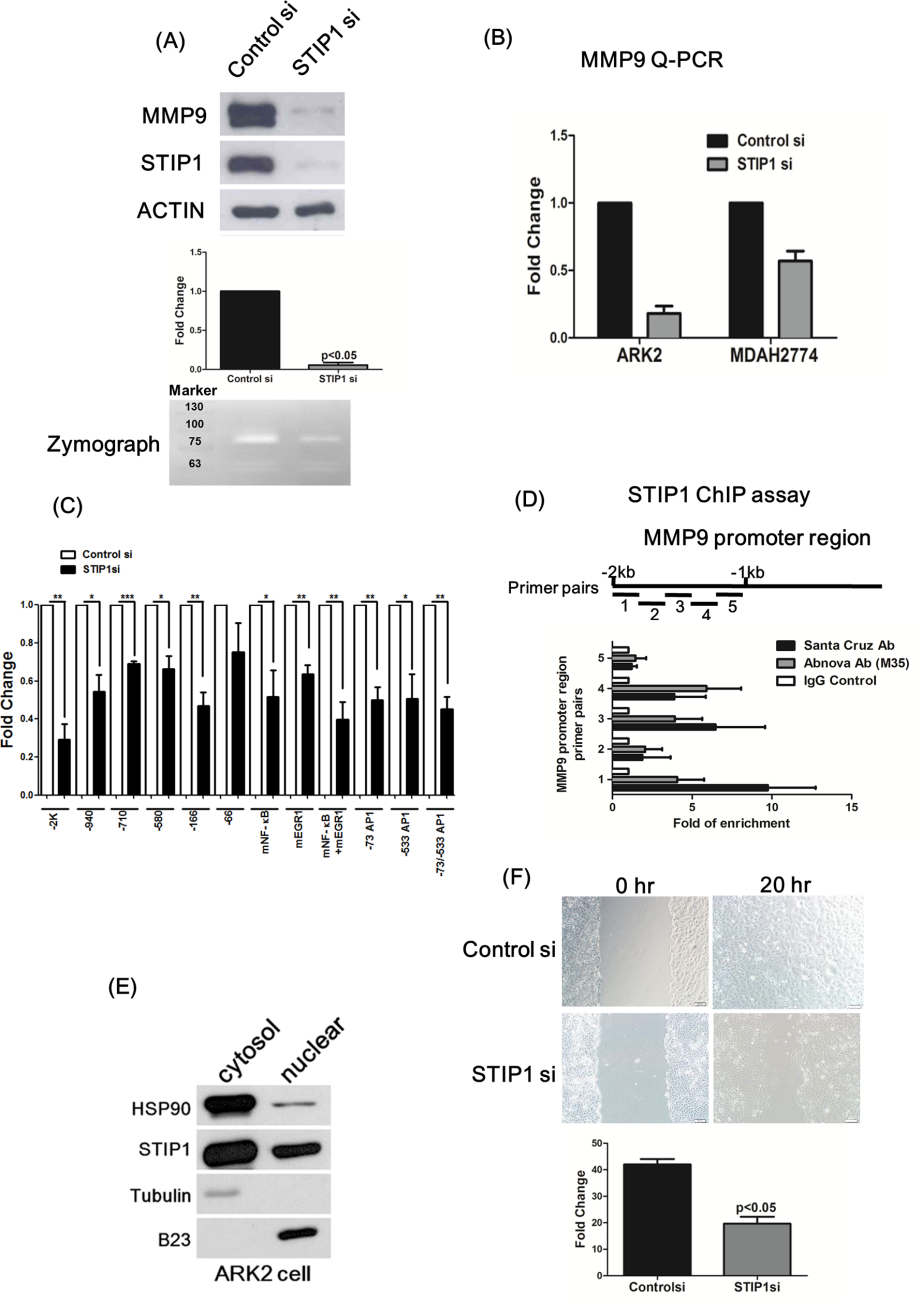
STIP1 binds to the MMP-9 promoter and is required for MMP-9 expression. (A) ARK2 cells were transfected with the control (scrambled) or STIP1 siRNAs. The protein levels of MMP-9, STIP1, and actin were analyzed using western blot. The bar graph summarizes the decreased protein levels of MMP9 that were caused by STIP1 knockdown in three independent siRNA experiments. A representative zymograph (lower panel) exemplifies the decreased MMP-9 activity that was caused by STIP1 knockdown. (B) At 72 h after transfection of the control or STIP1 siRNA, real-time quantitative polymerase chain reaction (RT-QPCR) was used to quantify MMP-9 RNA levels in the ARK2 and MDAH2774 cells. (C) ARK2 cells were transfected with various MMP-9 reporter constructs (described in the “Methods” section) in the presence of the control or STIP1 siRNA. Reporter activity was measured using the luciferase assay. Individual control (the empty bar for each reporter assay) indicated reporter activity in the cells cotransfected with the designated reporter construct and a control STIP1 siRNA, the activities of which were set as 1. Results are expressed as mean ± standard error from three independent experiments. Statistical differences were calculated with Student’s *t*-test. *p < 0.05, **p < 0.01, and ***p < 0.001. (D) Identification of STIP1 potential binding sites on the MMP-9 gene promoter (from −1 kb to −2 kb upstream of the transcriptional start site). Two commercially available antibodies (each obtained from Santa Cruz and Abnova) were used to pull-down STIP1. Three independent ChIP experiments were performed. Quantification was performed by RT-QPCR, with the results being expressed as mean ± standard error of the mean. (E) Nuclear/cytosolic fractionation of ARK2 cells was performed. HSP90, STIP1, tubulin, and B23 protein levels were quantified with western blot. Tubulin and B23 were used as cytosolic and nuclear markers, respectively. (F) Cell migration ability was blocked when STIP1 expression was silenced (quantification shown in the lower panel). Results are expressed as mean ± standard error of the mean from three independent experiments. Statistical differences were calculated using Student’s *t*-test.

Inhibition of the HSP90 chaperone machinery was recently shown to block MMP-9 expression [24]. The findings of a possible association between the expression levels of MMP-9 and STIP1 (Fig 1B) urged us to further evaluate the presence of a regulatory effect of STIP1 on MMP-9. Repression of STIP1 expression through siRNA blocked MMP-9 expression and activity in various cancer cells (Fig 2A and 2B).

The reporter activities of various MMP-9 promoter constructs (-2 kb, -940, -710, -580, and -166) were significantly suppressed by siRNA knockdown of STIP1, suggesting that STIP1 is involved in the transcriptional regulation of MMP-9 (Fig 2C). Although several transcription factors including NF-kB, EGR1, and AP1 regulate MMP-9 promoter activity, site-directed mutagenesis of transcriptional factor binding motifs in the MMP-9 promoter did not affect the inhibition of reporter activities by STIP1 knockdown, further indicating that STIP1 effects on MMP-9 stimulation did not occur through these known transcriptional factors (Fig 2C). We also used ChIP assays to test whether STIP1 physically interacted with the MMP-9 promoter. Because the −2 kb MMP-9 promoter reporter activities were repressed more efficiently by siRNA knockdown of STIP1 than the other constructs (Fig 2C), we designed five different primers (mean amplicon size, 200 bp) covering the MMP-9 promoter region (from −1 kb to −2 kb upstream of the transcriptional start site; Fig 2D). Two different commercially available antibodies (each from Santa Cruz and Abnova) were used to pull-down the STIP1 genomic DNA complex. The primer sets 1, 3, and 4 were capable of detecting STIP1 on the MMP-9 promoter (Fig 2D). Collectively, the results of reporter and ChIP assays indicate that STIP1 is involved in the transcriptional stimulation of MMP-9, and may, directly or indirectly, bind to the MMP-9 promoter.

The identification of STIP1 in both nuclear and cytoplasmic fractions indicated that STIP1 could be translocated into the nucleus to participate in the transcriptional regulation of MMP-9 expression (Fig 2E). Notably, wound-healing assays revealed suppression of cell migration following the knockdown of STIP1 expression (Fig 2F).

### Molecular mechanisms involved in the STIP1-mediated regulation of MMP-9

**Fig 3:**
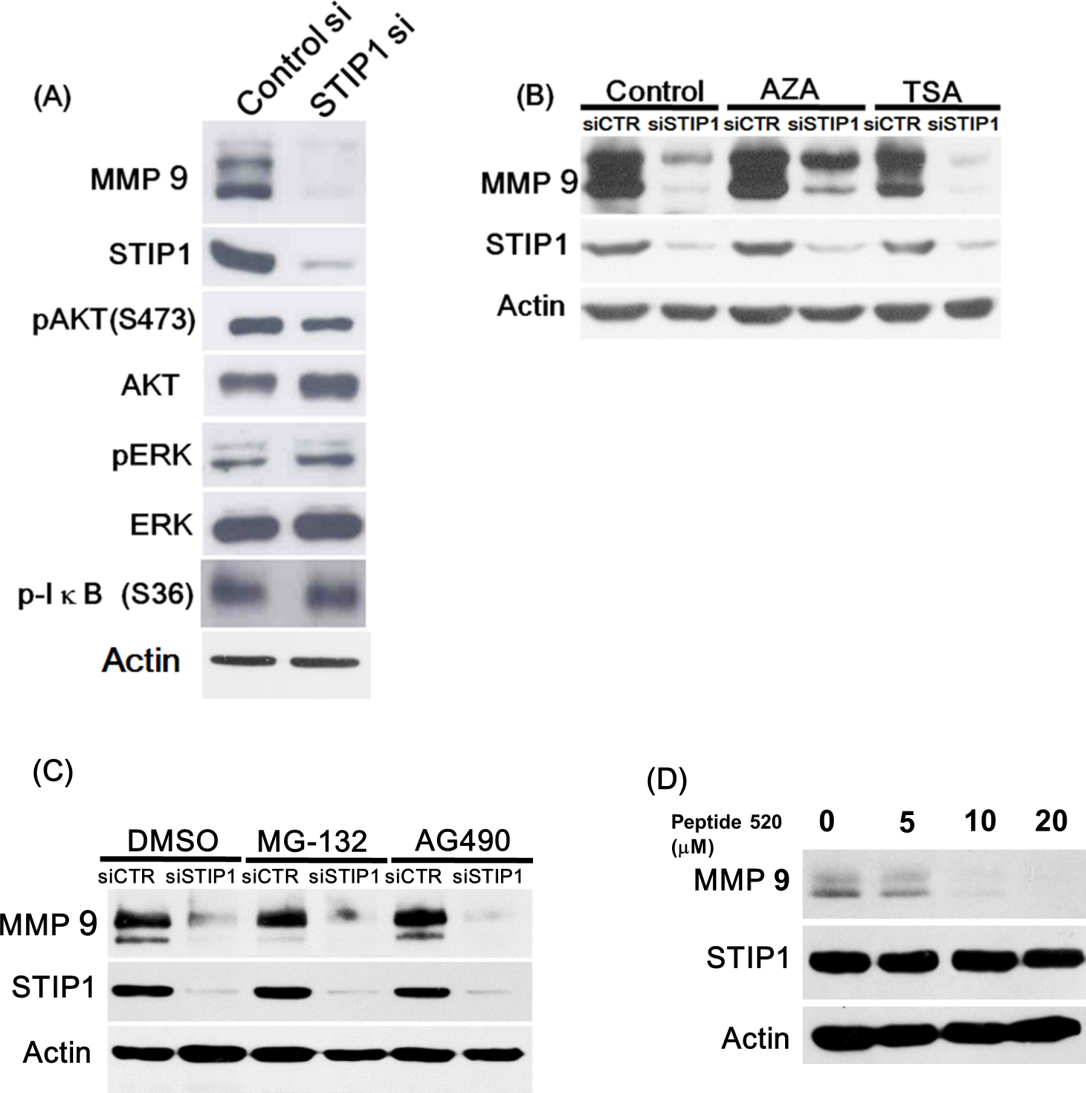
Decreased MMP-9 protein expression induced by STIP1 knockdown is independent of common signaling pathways. (A) STIP1 knockdown by siRNA in ARK2 cells decreased MMP-9 protein expression without affecting AKT, ERK, and IkB phosphorylation levels. (B) Decreased MMP-9 protein expression induced by siRNA knockdown of STIP1 was unaffected by exposure either to the DNA methylation inhibitor 5-aza-2’-deoxycytidine (AZA; 10 μM) or the histone deacetylase inhibitor trichostatin A (TSA; 20 nM). (C) Suppression of MMP-9 protein levels by siRNA knockdown of STIP1 was unaffected by treatment with either the proteasome inhibitor MG-132 (25 μM) or a JAK2 inhibitor AG490 (35 μM). (D) Treatment with an STIP1 inhibitor, peptide 520, suppressed MMP-9 protein levels in a dose-dependent manner.

STIP1 is one of the cochaperone proteins that form the structural parts of the HSP90 machinery. HSP90 inhibitors are capable of reducing MMP-9 expression via blunted NF-kB signaling [24]. Therefore, we investigated the role of the NF-kB pathway in STIP1-mediated regulation of MMP-9. Protein levels of phospho-AKT S473, phospho-ERK, and phospho-IkB S36 were not significantly altered following STIP1 suppression (Fig 3A). Furthermore, epigenetic modulation, including DNA methylation and histone modification, did not affect the STIP1-mediated regulation of MMP-9 as assessed by the use of specific inhibitors (Fig 3B). MMP-9 inhibition following STIP1 knockdown was not due to suppression of MMP-9 protein stability, because MMP-9 levels could not be restored by subsequent treatment with the proteasome inhibitor MG-132 (Fig 3C). We recently reported that STIP1 stabilizes JAK2 protein and that peptide 520—a peptide fragment in the DP2 domain of STIP1—can inhibit STIP1 functions [14]. However, the JAK2 inhibitor AG490 did not affect MMP-9 protein levels, excluding the involvement of the JAK2-STAT3 pathway in the regulation of MMP-9 (Fig 3C). On the other hand, treatment with the STIP1 inhibitor peptide 520 elicited a dose-dependent inhibition of MMP-9 protein levels (Fig 3D).

Taken together, the results in Figs 2 and 3 indicated that STIP1 was capable of binding to the MMP-9 promoter and enhancing its transcriptional expression. The transcriptional activation of MMP-9 by STIP1 was independent of NF-kB, ERK, AKT, and JAK2.

## Discussion

To the best of our knowledge, this is the first study to show that the upregulation of both STIP1 and MMP-9 is positively related to the pathophysiology of endometriosis/adenomyosis. Our findings are in line with a previous report where STIP1 was shown to stimulate the activity of MMPs [15]; moreover, several studies have indicated that MMP-9 is upregulated in endometriosis [16,18,19,25]. Although STIP1 has been found to be stimulated by estrogen [26], the findings of the present study point toward a theoretical framework in which the endometrial hyper-expression of STIP1 promotes extracellular matrix remodeling and degradation through increased MMP-9 activity. This could, in turn, enable the endometrial tissue to penetrate and migrate outside the uterus, ultimately resulting in the development of endometriosis. If this hypothesis is proven correct, STIP1 could be used as a promising therapeutic target for this condition.

The results of the present study suggest that STIP1 transcriptionally regulates the expression of MMP-9 (Fig 3); nevertheless, additional factors that may form the protein complex with STIP1 remain unidentified. Extracellular STIP1 [9,11,27] has been shown to suppress MMP-2 expression [15], exert cytokine-like activities [9], and activate SMAD-ID3 signaling pathways [11]. More recently, cytoplasmic STIP1 has been shown to maintain the stability of JAK2 protein [14]. Furthermore, STIP1 function can be inhibited by treatment with peptide 520 [14]. In the current study, the nontranscriptional regulation of MMP-9 by STIP1 via post-translational modification of proteins (methylation, acetylation) and several signaling pathways (AKT, ERK, NF-κB, and JAK2) has been ruled out based on the results summarized in Fig 3.

The main strength of our study is its comprehensive examination of the role of STIP1 in endometriosis through multiple experimental perspectives (i.e., assessment of its circulating levels, immunohistochemistry, and functional assays). However, our findings must be interpreted within the context of some limitations. First, primary stromal cells isolated from endometriotic tissues are hard to maintain in long-term culture and difficult to transfect. It is almost impossible to obtain consistent results from these cells in order to ascertain the molecular mechanisms that may exist in them. Therefore, we had to use endometrial cancer cells instead. Second, the usefulness of serum STIP1 levels for the diagnosis of endometriosis seems limited. However, advancements in new protein detection for STIP1 [28] may improve its clinical application.

In summary, our data suggest a potential role of STIP1 in endometriosis susceptibility, which may occur through the modulation of MMP-9 functions in a fashion independent of several known signaling proteins, such as NF-kB, ERK, AKT, and JAK2.

## Supporting information

S1 Fig Immunohistochemical (IHC) analyses of STIP1 and MMP9 in normal endometria. Positive IHC signals are stained in brown color. Scale bars represent 100 μm.
(DOCX)
