# Supplementary material for: Positive associations between upregulated levels of stress-induced phosphoprotein 1 and matrix metalloproteinase-9 in endometriosis/adenomyosis
Source: PLoS One. 2018 Jan 5;13(1):e0190573. doi: 10.1371/journal.pone.0190573 (PMC5755831; doi:10.1371/journal.pone.0190573)
Supplement: S1 Fig — Positive IHC signals are stained in brown color. Scale bars represent 100 μm. (DOCX) [file pone.0190573.s001.docx]

Supporting Information


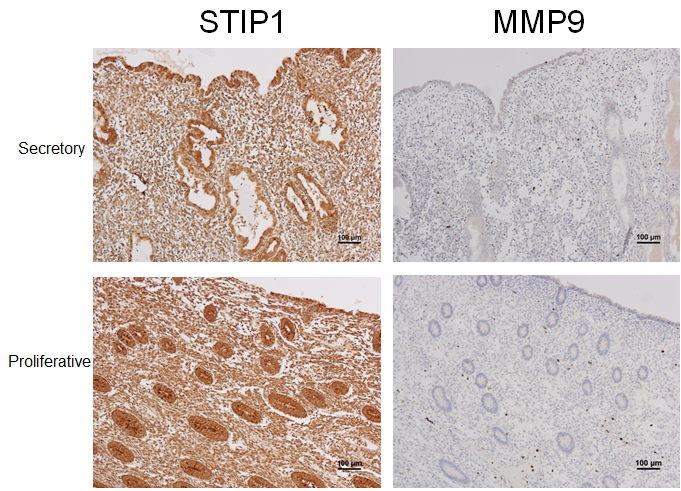


**S1 Fig.:Immunohistochemical (IHC) analyses of STIP1 and MMP9 in normal endometria.** Positive IHC signals are stained in brown color. Scale bars represent 100 μm.
